# Potentially inappropriate medication including drug-drug interaction and the risk of frequent falling, hospital admission, and death in older adults - results of a large cohort study (getABI)

**DOI:** 10.3389/fphar.2023.1062290

**Published:** 2023-02-15

**Authors:** Theresa Reinhild Haerig, Dietmar Krause, Renate Klaassen-Mielke, Henrik Rudolf, Hans Joachim Trampisch, Petra Thuermann

**Affiliations:** ^1^ Department of Medical Informatics, Biometry and Epidemiology, Ruhr University Bochum, Bochum, Germany; ^2^ Institute for Biostatistics and Informatics in Medicine and Ageing Research, University Medical Center Rostock, Rostock, Germany; ^3^ Philipp-Klee-Institute for Clinical Pharmacology, HELIOS Klinikum Wuppertal, University Witten/Herdecke, Wuppertal, Germany

**Keywords:** potentially inappropriate medication, drug-drug interaction, risk of frequent falling, hospital admission, mortality

## Abstract

**Introduction:** With growing age, multiple chronic diseases may result in polypharmacy. Drugs that should be avoided in older adults are called potentially inappropriate medications (PIM). Beyond PIM, drug-drug interactions (DDI) are known to be related to adverse drug events. This analysis examines the risk of frequent falling, hospital admission, and death in older adults associated with PIM and/or DDI (PIM/DDI) prescription.

**Materials and methods:** This *post hoc* analysis used data of a subgroup of the getABI study participants, a large cohort of community-dwelling older adults. The subgroup comprised 2120 participants who provided a detailed medication report by telephone interview at the 5-year getABI follow-up. The risks of frequent falling, hospital admission, and death in the course of the following 2 years were analysed by logistic regression in uni- and multivariable models with adjustment for established risk factors.

**Results:** Data of all 2,120 participants was available for the analysis of the endpoint death, of 1,799 participants for hospital admission, and of 1,349 participants for frequent falling. The multivariable models showed an association of PIM/DDI prescription with frequent falling (odds ratio (OR) 1.66, 95% confidence interval (CI) 1.06–2.60, *p* = 0.027) as well as with hospital admission (OR 1.29, 95% CI 1.04–1.58, *p* = 0.018), but not with death (OR 1.00, 95% CI 0.58–1.72, *p* = 0.999).

**Conclusion:** PIM/DDI prescription was associated with the risk of hospital admission and frequent falling. No association was found with death by 2 years. This result should alert physicians to provide a closer look at PIM/DDI prescriptions.

## 1 Introduction

With the improvement of living conditions and medical care, life expectancy is growing. Advanced age increases the risk of multimorbidity and polypharmacy that may cause drug-drug interactions (DDI) and adverse drug events (ADE), resulting in profound medical and safety problems for older adults and in economic effects on the healthcare system (Pohl-Dernick et al., 2016).

Drugs with an increased risk of potentially ADE for older adults are classified as potentially inappropriate medications (PIM) for this age group (Holt et al., 2010). Despite contradictory results, numerous studies have shown that PIM use is associated with an increased number of hospital admissions and – in some studies - also with higher morbidity and mortality (Reich et al., 2014; Henschel et al., 2015; Heider et al., 2017; Muhlack et al., 2017; Xing et al., 2019). In addition to PIM, DDIs are of great concern, being related to adverse drug reactions and frequent hospital stays (Johnell and Klarin, 2007; Xing et al., 2019). Almost half of preventable ADE is based on DDI, often occurring between antiplatelet drugs, oral anticoagulants, and non-steroidal anti-inflammatory drugs (NSAID). These drugs, as well as diuretics, have been identified as a frequent cause of preventable hospital admissions in older adults (Howard et al., 2007). The common combination of ACE-inhibitor or angiotensin receptor blocker plus diuretic plus NSAID was ascertained to be the unhappy triad resulting in deterioration of renal function (Lapi et al., 2013).

The first list with explicit criteria for PIMs was published 20 years ago (Beers 1991 criteria); the most recent update is reflected in the American Geriatrics Society Beers criteria (AGS Beers Criteria). The AGS Beers Criteria comprise an explicit list of PIMs that are typically best avoided by older adults in most circumstances or specific situations, for example with certain diseases or conditions (American Geriatics Society Beers Criteria^®^ Update Expert Panel, 2019). Due to differences in the drug market, PIM lists for older adults were published in different countries between 1991 and 2017 (Motter et al., 2018). The European Union (EU) (7)-PIM list is a screening tool, developed with participation of experts from seven European countries that allows identification and comparison of PIM prescribing patterns for older adults across European countries (Renom-Guiteras et al., 2015). For the German drug market, the PRISCUS list has been established in 2010 (Holt et al., 2010). The PRISCUS list includes 18 drug groups with a total of 83 drugs and has been applied for several pharmaco-epidemiological analyses as well as for prospective studies.

Some contradictory results regarding the association of PIM use with adverse outcomes have been published (Xing et al., 2019). This may be a result of different populations studied (community-dwelling seniors *versus* nursing home residents), but also an effect of different methods to estimate drug exposure. Many studies used prescription and claims data to calculate exposure and to detect adverse outcomes. The present study aims to analyse the risk of frequent falling, hospital admission, and death associated with PIM/DDI prescription in a prospective cohort study observing community-dwelling older persons with regular follow-ups including patients’ interviews. PIM was defined by the PRISCUS list (Holt et al., 2010) and DDI according to the study “Reduction of potentially Inappropriate Medication in the Elderly” (RIME) (Rudolf et al., 2021). Data about medication use were available from direct patient reports.

## 2 Materials and methods

The basis of the present study is the getABI study that was set up to obtain reliable data on the epidemiology, comorbidities, and risk factor profile of peripheral arterial disease (PAD) in general medical practices and has been described elsewhere (Diehm et al., 2004). It started in 2001 in Germany and included unselected primary care patients at the age of 65 years or older who were observed for 7 years (getABI study group, 2002). At getABI-baseline, general and medical history were obtained, a physical examination including ankle-brachial index (ABI) measurement and blood-sampling were performed. Follow-up visits with ABI measurements and physicians’ assessments, documented in a case report form (CRF) took place after one, three, five, and 7 years. The 5-year and 7-year follow-up additionally comprised patients’ reports with a detailed listing of medications and questionnaires, e. g. concerning health-related quality of life (HRQOL), obtained by telephone interviews. A standardized assessment on information about name, pharmaceutical central number, dose, and the pharmaceutical form of medication was conducted by trained interviewers.

All patients with an available interview at the 5-year getABI follow-up were included in this analysis. For this *post hoc* analysis we labelled the 5-year getABI follow-up as ‘PIM/DDI baseline’, where study endpoints were taken from the 7-year follow-up.

‘Frequent falling’ defined as 2 or more falls per year was recorded retrospectively 2 years after PIM/DDI baseline for the preceding 12 months. Hospital admission was determined by the CRF 2 years after PIM/DDI baseline for the preceding 2 years. The vital status was also taken by the CRF. If there was no information in the CRF and the telephone interview was missing, the vital status was requested at the civil registration office.

As secondary endpoint, HRQOL was measured by the three-level version of the EuroQol five-dimensional questionnaire (EQ-5D-3L) (Fuller et al., 2017). We dichotomized the data of the EQ-5D-3L at the 25th percentile of the age- and sex-adjusted general population of Germany and labelled values from the lowest quarter ‘low HRQOL’ (Szende et al., 2014). The secondary endpoint was defined by low HRQOL as documented during the interview 2 years after PIM/DDI baseline.

All medication data were coded according to the German Anatomical Therapeutical Chemical (ATC)-code in the version of 2006 (Fricke et al., 2019). Combination preparations were counted according to the number of active ingredients. There were two categories of medication risk factors of interest recorded at PIM/DDI baseline:

1) PIM according to the PRISCUS list (Holt et al., 2010);
2) Four DDIs, as defined in the study ‘Reduction of potentially Inappropriate Medication in the Elderly’ (RIME) (Rudolf et al., 2021):
  - antiplatelet drug/oral anticoagulation plus non-steroidal anti-inflammatory drug (NSAID) without co-prescription of a proton pump inhibitor (PPI),
  - multiple antiplatelet drugs without PPI,
  - antiplatelet drug plus oral anticoagulation without PPI,
  - ACE-inhibitors/AT1-antagonists plus NSAIDs.

In this analysis, PIM and/or DDI prescriptions are labelled as ‘PIM/DDI’.

Other risk factors taken into account were age, male sex, low education as defined by an International Standard Classification of Education (ISCED) score of 0–2, and smoker status, taken at baseline of the getABI cohort study. Arterial hypertension was either diagnosed at getABI baseline or defined as use of antihypertensive medication at getABI baseline or at the PIM/DDI baseline. Likewise, information about diabetes was either taken from the getABI baseline (HbA1c > 47.5 mmol/mol) or defined as use of antidiabetic medication at any time point. From the baseline investigation of the getABI study information was taken about cholesterol levels. Peripheral artery disease was defined by ABI <0.9 (ABI >1.5 was labelled mediasclerosis) or in case of symptoms/events like peripheral revascularisation, necrosis, gangrene, intermittent claudication, or amputation before PIM/DDI baseline. Impaired renal function was defined as eGFR <60 ml/min/1.73 m^2^ measured before PIM/DDI baseline.

### 2.1 Statistical methods

The characteristics of the participants at PIM/DDI baseline (including getABI baseline data as mentioned above) are presented as means and standard deviations or numbers and percentages, as appropriate. Differences between the PIM/DDI group and the non-PIM/DDI group were analysed using the *t*-test or the chi-square-test, respectively; *p*-values are displayed.

Logistic regression was applied in univariable and multivariable models. For all the endpoints we used univariable and multivariable models including the above-mentioned risk factors. Missing values of risk factor data were randomly replaced by values of the entire cohort up to a proportion of 2%.

The results were presented as odds ratios (OR) with 95% confidence interval (CI). We used two-sided *p*-values and labelled *p*-values <0.05 as significant. Analyses were performed using SAS, version 9.4 (2013, SAS Institute Inc., Cary, NC, United States).

The institutional review board of the University of Heidelberg had approved the getABI trial. Each patient had provided written informed consent. The getABI trial followed the recommendations of Good Epidemiological Practice and was supported by unrestricted grants from Sanofi-Aventis GmbH, Berlin, Germany, and the German Federal Ministry of Education and Research. The protocol of this *post hoc* analysis was reviewed and approved by the ethics committee of the Ruhr University Bochum, Germany (approval number: 17-6103); the data were analysed anonymously. Trial registration DRKS00014098.

## 3 Results

**FIGURE 1:**
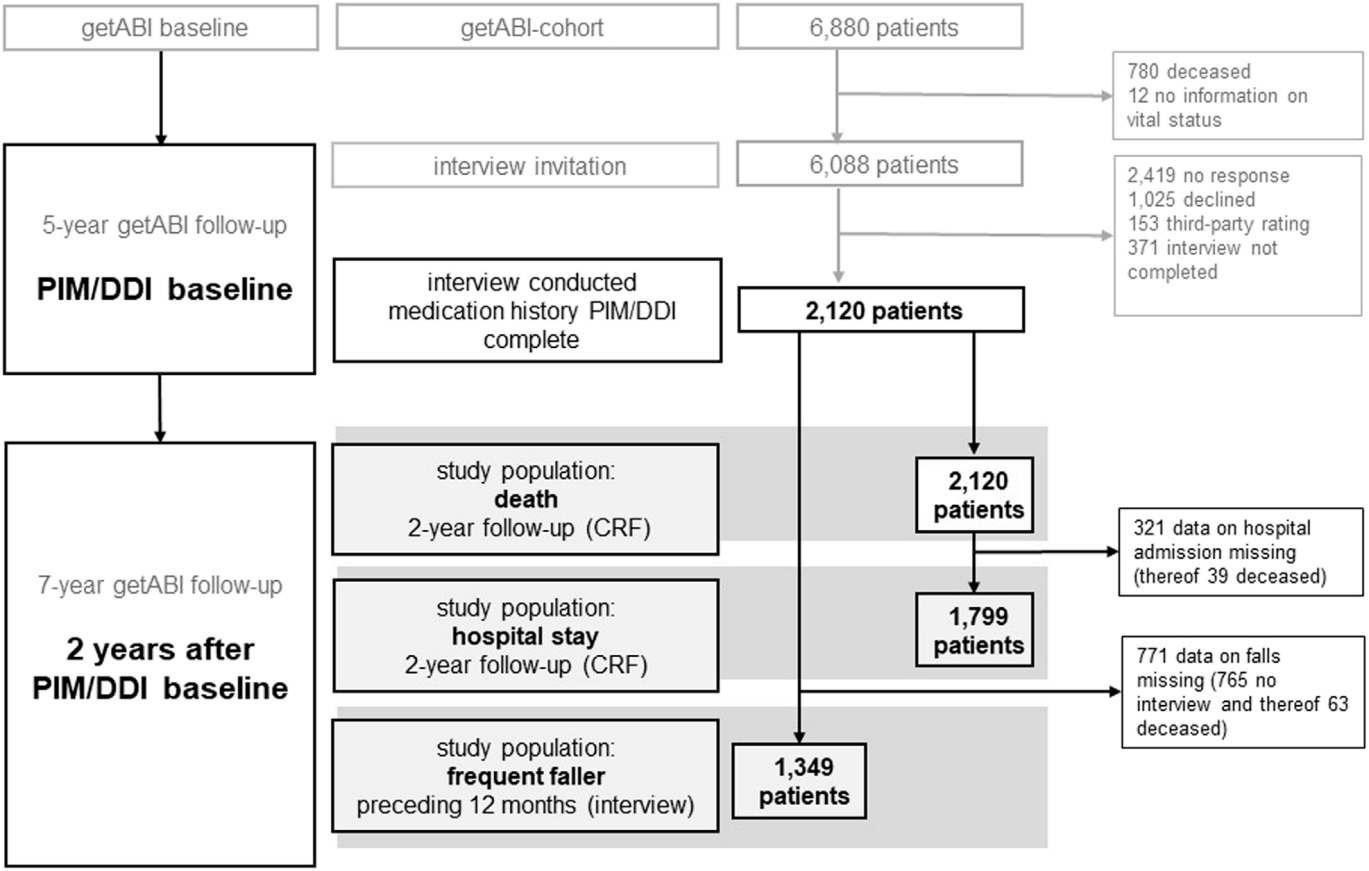
Flow chart outline of the inclusion of participants in the analyses.

The getABI cohort comprised 6,880 participants at the getABI baseline. At the 5-year getABI follow-up (PIM/DDI baseline), 6,088 of them were still alive and 2,120 gave written informed consent to a telephone interview. These patients made up the cohort of this *post hoc* analysis (Figure 1). Two years later, sufficient information concerning survival could be obtained from all 2,120 patients, concerning hospital admission from 1,799 (84.9%) patients, and from 1,349 (63.6%) patients for frequent falling (Figure 1).

At PIM/DDI baseline, 441 (20.8%) of these 2,120 patients had at least one PIM, 359 (16.9%) at least one DDI, and 685 patients (32.3%) made up the PIM/DDI subgroup. The mean age of all 2,120 patients was 76 years, 54% were women, and 77% had arterial hypertension, with nearly 87% in the PIM/DDI subgroup. Peripheral artery disease (PAD), myocardial infarction, stroke, kidney diseases/impaired renal function, and a significantly lower HRQOL were more often found in the PIM/DDI subgroup (Table 1).

**TABLE 1:** Baseline characteristics of study participants.

Variable	Missing values	PIM and/DDI (n = 685)	No PIM and no DDI (n = 1,435)	Total (n = 2,120)	*p*-Value
Age, years, mean (sd)	0	76.8 (4.7)	76.0 (4.3)	76.3 (4.5)	<0.001
Female n (%)	0	381 (55.6)	757 (52.8)	1138 (53.7)	0.216
ISCED^1, 2^ low (0-2), n (%)	0	118 (17.2)	232 (16.2)	350 (16.5)	0.539
Increased waist hip ratio^1^, n (%)	0	544 (79.4)	1088 (75.8)	1632 (77.0)	0.066
Smoker^1^ (current), n (%)	0	46 (6.7)	104 (7.2)	150 (7.1)	0.655
Arterial hypertension^3^, n (%)	0	595 (86.9)	1039 (72.4)	1634 (77.1)	<0.001
Diabetes^3, 4^, n (%)	0	183 (26.7)	348 (24.3)	531 (25.0)	0.221
Elevated CRP^1^ ( ≥ 3 mg/L), n (%)	0	265 (38.7)	477 (33.2)	742 (35.0)	0.014
LDL^1^ ≥ 130 mg/dl, n (%)	0	282 (41.2)	630 (43.9)	912 (43.0)	0.234
Statin/fibrate use^3^, n (%)	0	279 (40.7)	499 (34.8)	778 (36.7)	0.008
Peripheral arterial disease^3^, n (%)	0	268 (39.1)	438 (30.5)	706 (33.3)	<0.001
Depression likely, n (%)	128	32 (5.0)	36 (2.7)	68 (3.4)	0.007
Myocardial infarction^3^, n (%)	0	86 (12.6)	130 (9.1)	216 (10.2)	0.013
Stroke^3^, n (%)	0	57 (8.3)	77 (5.4)	134 (6.3)	0.009
Kidney disease or impaired renal function^3^, n (%)	0	141 (20.6)	225 (15.7)	366 (17.3)	0.005
Polypharmacy^3^ ( ≥ 5 drugs), n (%)	0	459 (67.0)	465 (32.4)	924 (43.6)	<0.001
Health related quality of life^3^ (EQ-5D-3L raw), mean (sd)	17	73.0 (15.7)	80.1 (14.6)	77.8 (15.3)	<0.001

^1^getABI baseline (not available at PIM/DDI baseline).

^2^International Standard Classification of Education ISCED).

^3^according to history up to PIM/DDI baseline.

^4^Type 1 or Type 2.

The endpoint death was met by 63 (3.0%) participants, 22 (3.2%) in the PIM/DDI group and 41 (2.9%) in the non-PIM/DDI group (Table 2). No significant effect on mortality could be shown for the risk factor PIM/DDI (multivariable model: odds ratio (OR) 1.00, confidence interval (CI) 0.58–1.72, *p* = 0.999).

**TABLE 2:** Univariable models and multivariable model for the endpoint death (2,120 patients).

Variable	Odds ratio [95%-CI] univariable	*p*-Value univariable	Odds ratio [95%-CI] multivariable	*p*-Value multivariable
Age (per year)	1.10 [1.04; 1.16]	<0.000	1.10 [1.04; 1.17]	0.001
Sex (male vs. female)	2.38 [1.40; 4.04]	0.001	2.70 [1.46; 4.97	0.001
Education level^1, 2^ (low vs. middle/high)	1.33 [0.71; 2.47]	0.371	1.90 [0.93; 3.88]	0.077
Arterial hypertension^3^ (yes vs. no)	1.27 [0.67; 2.41]	0.459	0.94 [0.47; 1.85]	0.849
Diabetes^3, 4^ (yes vs. no)	1.52 [0.89; 2.59]	0.126	1.29 [0.74; 2.25]	0.370
Smoker^1^ (current vs. former or no)	1.40 [0.59; 3.30]	0.444	1.32 [0.54; 3.24]	0.548
LDL ≥ 130 mg/dL^1^ (yes vs. no)	0.56 [0.33; 0.97]	0.039	0.65 [0.37; 1.13]	0.129
Myocardial infarction^3^ (yes vs. no)	3.16 [1.76; 5.68]	0.000	2.32 [1.23; 4.37]	0.010
Stroke^3^ (yes vs. no)	0.23 [0.03; 1.70]	0.150	0.16 [0.02; 1.16]	0.070
Kidney disease or impaired renal function^3^ (yes vs. no)	1.13 [0.60; 2.14]	0.704	0.65 [0.32; 1.32]	0.236
Peripheral arterial disease^3^ (yes vs. no)	2.42 [1.46; 4.00]	0.001	2.00 [1.17; 3.44]	0.012
PIM/DDI^3^ (yes vs. no)	1.13 [0.67; 1.91]	0.653	1.00 [0.58; 1.72]	0.999

^1^getABI baseline (not available at PIM/DDI baseline).

^2^International Standard Classification of Education (ISCED).

^3^PIM/DDI baseline.

^4^Type 1 or Type 2.

721 (40.1) out of 1,799 participants had hospital stays (255 in the PIM/DDI group (45.2%), 466 in the non-PIM/DDI group (37.7%)). In the univariable model, the prescription of PIM/DDI was significantly associated with hospital admission (OR 1.36, 95% CI 1.11–1.67, *p* = 0.003). In the multivariable model with adjustment for age, sex, and other risk factors, PIM/DDI prescription showed an OR of 1.29 (95% CI 1.04–1.58, *p* = 0.018) (Table 3).

**TABLE 3:** Univariable models and multivariable model for the endpoint hospital admission (1,799 patients).

Variable	Odds ratio [95%-CI] univariable	*p*-value univariable	Odds ratio [95%-CI] multivariable	*p*-value multivariable
Age (per year)	1.04 [1.02; 1.06]	0.001	1.03 [1.01; 1.05]	0.009
Sex (male vs. female)	1.07 [0.89; 1.30]	0.454	1.08 [0.88; 1.32]	0.475
Education level^1, 2^ (low vs. middle/high)	1.14 [0.88; 1.47]	0.320	1.15 [0.88; 1.51]	0.308
Arterial hypertension^3^ (yes vs. no)	1.21 [0.96; 1.51]	0.106	1.04 [0.82; 1.33]	0.735
Diabetes^3, 4^ (yes vs. no)	1.17 [0.94; 1.45]	0.150	1.13 [0.90; 1.41]	0.302
Smoker^1^ (current vs. former or no)	1.36 [0.94; 1.97]	0.098	1.43 [0.98; 2.09]	0.061
LDL^1^ ≥ 130 mg/dL y0 (yes vs. no)	1.06 [0.87; 1.28]	0.574	1.11 [0.92; 1.35]	0.284
Myocardial infarction^3^ (yes vs. no)	1.61 [1.18; 2.18]	0.003	1.47 [1.06; 2.03]	0.021
Stroke^3^ (yes vs. no)	1.48 [1.02; 2.16]	0.041	1.30 [0.88; 1.92]	0.181
Kidney disease or impaired renal function^3^ (yes vs. no)	1.27 [0.99; 1.63]	0.056	1.08 [0.83; 1.41]	0.573
Peripheral arterial disease^3^ (yes vs. no)	1.38 [1.13; 1.69]	0.002	1.20 [0.97; 1.48]	0.093
PIM/DDI^3^ (yes vs. no)	1.36 [1.11; 1.67]	0.003	1.29 [1.04; 1.58]	0.018

^1^getABI baseline (not available at PIM/DDI baseline).

^2^International Standard Classification of Education (ISCED).

^3^PIM/DDI baseline.

^4^Type 1 or Type 2.

98 (7.3%) out of 1,349 participants were frequent fallers (43 in the PIM/DDI group (10.2%), 55 in the non-PIM/DDI group (5.9%)). In the univariable model, the prescription of PIM/DDI was significantly associated with frequent falling (OR 1.80, 95% CI 1.19–2.73, *p* = 0.006). In the multivariable model with adjustment for age, sex, and other risk factors, PIM/DDI prescription showed an OR of 1.66 (95% CI 1.06–2.60, *p* = 0.027) (Table 4).

**TABLE 4:** Univariable models and multivariable model for the endpoint frequent falling (1,349 patients).

Variable	Odds ratio [95%-CI]univariable	*p*-value univariable	Odds ratio [95%-CI]multivariable	*p*-value multivariable
Age (per year)	1.05 [1.00; 1.10]	0.039	1.05 [0.99; 1.10]	0.089
Sex (male vs. female)	0.60 [0.39; 0.92]	0.020	0.53 [0.33; 0.86]	0.010
Education level^1, 2^ (low vs. middle/high)	1.32 [0.76; 2.28]	0.324	1.18 [0.66; 2.11]	0.573
Arterial hypertension^3^ (yes vs. no)	1.33 [0.79; 2.26]	0.283	1.03 [0.57; 1.83]	0.932
Diabetes^3, 4^ (yes vs. no)	1.31 [0.84; 2.05]	0.236	1.43 [0.89; 2.31]	0.143
Smoker^1^ (current vs. former or no)	0.29 [0.07; 1.21]	0.090	0.43 [0.10; 1.80]	0.246
LDL^1^ ≥ 130 mg/dl (yes vs. no)	0.68 [0.44; 1.05]	0.081	0.70 [0.45; 1.11]	0.129
Myocardial infarction^3^ (yes vs. no)	1.54 [0.85; 2.80]	0.154	1.83 [0.95; 3.51]	0.069
Stroke^3^ (yes vs. no)	1.71 [0.85; 3.42]	0.130	1.51 [0.70; 3.23]	0.291
Kidney disease or impaired renal function^3^ (yes vs. no)	1.21 [0.72; 2.02]	0.469	1.01 [0.57; 1.78]	0.981
Peripheral arterial disease^3^ (yes vs. no)	0.89 [0.56; 1.42]	0.624	0.75 [0.45; 1.25]	0.263
PIM/DDI^3^ (yes vs. no)	1.80 [1.19; 2.73]	0.006	1.66 [1.06; 2.60]	0.027

^1^getABI baseline (not available at PIM/DDI baseline).

^2^International Standard Classification of Education (ISCED).

^3^PIM/DDI baseline.

^4^Type 1 or Type 2.

Considering the secondary endpoint low HRQOL, 1,322 (62.4%) participants provided sufficient data for the analysis. 336 (25.4%) out of 1,799 participants reported low HRQOL (139 in the PIM/DDI group (33.7%), 197 in the non-PIM/DDI group (21.6%)). In the univariable model, the OR of PIM/DDI for this endpoint was 1.85 (95% CI 1.43–2.39, *p* < 0.001). The OR remained significant in the multivariable model (1.40 (95% CI 1.04–1.88, *p* = 0.027). Thus, despite adjustment for the PIM/DDI baseline data (and other risk factors), PIM/DDI prescription was associated with low HRQOL after 2 years (Table 5).

**TABLE 5:** Univariable models and multivariable model for the secondary endpoint lowHROQL 2 years after PIM/DDI baseline measured by the EQ-5D-3L (1,322 patients).

Variable	Univariable		Multivariable	
	Odds Ratio [95%-CI]	*p*-value	Odds Ratio [95%-CI]	*p*-value
Health related quality of life (EQ-5D-3L) (low vs. others)	7.71 [5.86; 10.1]	<0.001	7.48 [5.59; 9.99]	<0.001
Age (per year)	0.95 [0.92; 0.98]	0.001	0.97 [0.94; 1.01]	0.153
Sex (male vs. female)	1.20 [0.94; 1.54]	0.143	0.89 [0.64; 1.23]	0.477
Education level^1, 2^ (low vs. middle/high)	1.19 [0.84; 1.67]	0.335	1.34 [0.89; 2.02]	0.157
Arterial hypertension^3^ (yes vs. no)	1.47 [1.08; 2.01]	0.015	1.18 [0.82; 1.70]	0.380
Diabetes^3, 4^ (yes vs. no)	1.50 [1.14; 1.97]	0.003	1.19 [0.87; 1.64]	0.279
Smoker^1^ (current vs. former or no)	1.06 [0.83; 1.35]	0.655	0.92 [0.68; 1.26]	0.611
LDL^1^ ≥ 130 mg/dl (yes vs. no)	0.88 [0.68; 1.13]	0.307	0.94 [0.71; 1.25]	0.674
Myocardial infarction^3^ (yes vs. no)	1.67 [1.15; 2.44]	0.008	1.48 [0.94; 2.33]	0.088
Stroke^3^ (yes vs. no)	1.62 [1.03; 2.55]	0.038	1.73 [1.02; 2.93]	0.042
Kidney disease or impaired renal function^3^ (yes vs. no)	1.24 [0.90; 1.69]	0.182	1.11 [0.77; 1.61]	0.582
Peripheral arterial disease^3^ (yes vs. no)	1.28 [0.98; 1.67]	0.069	1.12 [0.82; 1.53]	0.476
PIM/DDI^3^ (yes vs. no)	1.85 [1.43; 2.39]	<0.001	1.40 [1.04; 1.88]	0.027

^1^getABI baseline (not available at PIM/DDI baseline).

^2^International Standard Classification of Education (ISCED).

^3^PIM/DDI baseline.

^4^Type 1 or Type 2.

## 4 Discussion

This *post hoc* analysis comprised 2,120 participants 70 years or older in a primary care setting, 685 of them (32.3%) had a PIM or a DDI. This PIM/DDI prevalence is comparable to other German and international studies. In one of them based on health insurance data of more than 800.000 persons at least 65 years old, 25.0% received at least one PIM prescription (Amann et al., 2012). A German claims data analysis with 73,665 insurants of the same age group yielded a PIM prevalence of 22% (Schubert et al., 2013). In the German health interview and examination survey (DEGS1) only 13% were PIM users (Endres et al., 2017). In contrast, in a cohort of elderly Austrian primary care patients with polypharmacy, a PIM prevalence of 37% was found (Koper et al., 2013). These data correspond well with the prevalence of PIM use observed in the US population of community dwelling seniors ranging between 11 and 51% (Nothelle et al., 2019) as well with the prevalence in European older citizens ranging calculated with 22.6% (Tommelein et al., 2015). Differences in PIM prevalence can be explained by the different populations analysed, criteria applied, the sources of information used and the methods to calculate the prevalence (Xing et al., 2019). Irrespective of these differences, the actual taking of these drugs is not proven in any study. We used patients’ interviews and thus documented patients’ statements rather than claims data. However, patient reports on medication use as well as health outcomes depend on social desirability, cognitive function and recall bias.

In our analysis, the endpoint death was not significantly associated with the use of PIMs or occurrence of the three selected DDIs. PIM use as defined by the Beers criteria or the HEDIS-DAE list was associated with 1.6-fold increased mortality in older adults (Muhlack et al., 2017). Heider et al. applied the PRISCUS list to a German claims database where approximately 500.000 PIM users were matched to almost four million non-users. PIM use was associated with a 1.8-fold higher risk of mortality compared to non-users (Heider et al., 2017). However, most studies also failed to demonstrate a higher odds ratio for death under PIM use (Mekkonnen et al., 2021). Interestingly, in one meta-analysis, the association between PIM use and mortality became significant when considering only studies with a new user design indicating an increased risk particularly at the initiation of PIM therapy (Xing et al., 2019). Moreover, the significance of the association between PIM use and adverse outcomes may also be influenced by the overall prescribing prevalence of PIM (Xing et al., 2019). Furthermore, the negative results for the endpoint mortality may also be explained by the fact, that the complexity of the telephone interview and the medication questionnaire might have resulted in a selection of healthier participants in this *post hoc* study.

Inappropriate medication use in older multimorbid people is associated with a range of negative healthcare consequences including adverse drug events and unplanned hospitalizations (Xing et al., 2019; Mekkonnen et al., 2021). In our study, PIM/DDI use was associated with a significant higher rate of hospitalisations (OR = 1.29; 95% CI 1.04-1.58). Another study, also using the PRISCUS list, showed that PIM use compared to PIM alternatives was associated with an increased risk of all-cause hospitalization in the 180 days following individual index date (OR = 1.38; 95% CI: 1.35–1.41) (Endres et al., 2016). In a 5-year prospective cohort study with 196 patients using modified Beers Criteria, PIMs were correlated with first hospitalization (HR 1.91, 95% CI 1.17-3.09) (Huang et al., 2019). 647,073 patients aged ≥65 years with PIM were compared to matched patients without PIM. The OR for hospitalization was 1.54 [95% CI 1.23–1.93] for PIM patients compared to non-PIM patients (Henschel et al., 2015). In an elderly managed care population in Switzerland, multiple cox regression analysis revealed a significant association with adverse outcomes in terms of hospitalizations (Reich et al., 2014).

98 (7.3%) out of 1,349 participants in our study were frequent fallers (43 in the PIM/DDI group (10.2%), 55 in the non-PIM/DDI group (5.9%)), yielding an OR of 1.66 in the multivariable model. Bauer et al. used the PRISCUS list to analyse the risk of fall-related injuries in a sample of frail patients above the age of 65 years. In their cohort, use of psychotropics and/or drugs from the PRISCUS list was significantly associated with fall-related injuries derived from administrative data (Bauer et al., 2012). A correlation between falls and PIMs, as well as the class of medication, was seen in community-dwelling older adults aged 55 and older (Lawson et al., 2018). Also, in a retrospective study with 667 patients, PIM use as defined by the Beers criteria was associated with the risk of falls (OR 2.24, 95% CI 1.51-3.32) (Kucugdagli et al., 2020). In their meta-analysis, Mekkonen et al. reported a significant association between PIM use and falls (Mekkonnen et al., 2021). However, as emphasized by Bauer et al. (Bauer et al., 2012), not all drugs from the PRISCUS list are specific for fall risk and so-called fall-risk increasing drugs (FRIDS) (Seppala et al., 2018), some of which are on the PRISCUS list (e.g. long-acting benzodiazepines) and are closer correlated with falls (Leipzig et al., 1999; Dhalwani et al., 2017). Unfortunately, we did not differentiate between PIM subclasses with a special subgroup of PIM-FRIDS.

Furthermore, our study shows that PIM/DDI prescription may be associated with a reduced HRQOL. An American study investigated the relationship between two widely used generic HRQOL measures (Short Form-12 (SF-12) and EQ-5D-3L) and potentially inappropriate drug use in a cohort of higher aged people (Franic and Jiang, 2006). PIM use was not a significant predictor of HRQOL in any of the models tested, but the number of prescriptions was a significant predictor of HRQOL, as measured by using the SF-12 and the EQ-5D. The same was found in hospitalized older patients (Akkawi et al., 2019). The contradictory results reported in the literature (Mekkonnen et al., 2018) may rely on different methodological approaches, but also on the fact that many drugs on the PIM lists are psychotropics and analgesics and such results might be confounded by indication.

Although our study, as many others (Mekkonnen et al., 2018) show significant associations between PIM use, certain DDIs and adverse outcomes, it is still unclear whether reduction of PIMs cleary results in improvements of outcomes. Recent systematic reviews stated that even any effect of interventions to improve the appropriate use of polypharmacy for older people is rather small and there is no evidence for reduction of unwanted outcomes such as hospital admissions, morbidity, and mortality as well as improvement of QoL (Rankin et al., 2018; Anderson et al., 2020). However, several analyses of claims data as well as cohort studies from Germany suggest a substantial decrease of PIM prevalence during the last 10 years (Zimmermann et al., 2013; Selke-Krulichova et al., 2021). It is unclear, if this is the result of increased awareness after the publication of the PRISCUS list in 2010, or a generally higher recognition of problematic prescribing in older patients supported by an increase in seminars and lectures. In addition, the PRISCUS list has been adopted in most major electronic prescribing tools in Germany. As interdisciplinary teams involving clinical pharmacists, some of them specialised in geriatric pharmacy, are getting more and more into practice, this may also contribute to safer prescribing (Moecker et al., 2022).

This study has some limitations. We did not capture the changes in medication and PIM/DDI status during the 2-year follow-up period. In addition, we limited our DDI definition to 4 major interactions and disregarded other possible DDIs (de Oliveira et al., 2021). No differentiation was made between PIM subclasses, nor was any differentiation made with respect to causes of hospitalization or death. Data on hospitalizations were missing for 282 patients and on frequent falls for 771 patients (of whom 63 died). Restricting 6,088 getABI patients who were still alive to the 2,120 participants in this study with written informed consent for telephone interview could introduce substantial selection bias.

A strength of this study is that data about medication use was available from direct patient reports increasing the probability of actual intake and that there was a direct assessment of the quality of life by telephone interviews. A further strength is the joint recording of PIM and DDI according to the RIME study.

In summary, our results show that PIM/DDI prescription may have an impact on hospital admission and frequent falling and may be associated with a reduction of health-related quality of life. As an aging society bears the burden of an increasing rate of chronic diseases and polypharmacy, the development of effective tools to improve medication management is urgently needed.

## Data Availability

The original contributions presented in the study are included in the article/Supplementary materials, further inquiries can be directed to the corresponding author.
